# Novel roles for scleraxis in regulating adult tenocyte function

**DOI:** 10.1186/s12860-018-0166-z

**Published:** 2018-08-07

**Authors:** Anne E. C. Nichols, Robert E. Settlage, Stephen R. Werre, Linda A. Dahlgren

**Affiliations:** 1 Department of Large Animal Clinical Sciences, Virginia-Maryland College of Veterinary Medicine, Virginia Tech, 205 Duck Pond Drive, Blacksburg, VA 24061-0442 USA; 2 Advanced Research Computing, Virginia Biocomplexity Institute, Virginia Tech, Blacksburg, VA 24061 USA; 3 Laboratory for Study Design and Statistical Analysis, Virginia-Maryland College of Veterinary Medicine, Blacksburg, VA 24061 USA

**Keywords:** Tendon, Tenocyte, Scleraxis, Mechanotransduction, RNA-seq

## Abstract

**Background:**

Tendinopathies are common and difficult to resolve due to the formation of scar tissue that reduces the mechanical integrity of the tissue, leading to frequent reinjury. Tenocytes respond to both excessive loading and unloading by producing pro-inflammatory mediators, suggesting that these cells are actively involved in the development of tendon degeneration. The transcription factor scleraxis (Scx) is required for the development of force-transmitting tendon during development and for mechanically stimulated tenogenesis of stem cells, but its function in adult tenocytes is less well-defined. The aim of this study was to further define the role of Scx in mediating the adult tenocyte mechanoresponse.

**Results:**

Equine tenocytes exposed to siRNA targeting Scx or a control siRNA were maintained under cyclic mechanical strain before being submitted for RNA-seq analysis. Focal adhesions and extracellular matrix-receptor interaction were among the top gene networks downregulated in Scx knockdown tenocytes. Correspondingly, tenocytes exposed to Scx siRNA were significantly softer, with longer vinculin-containing focal adhesions, and an impaired ability to migrate on soft surfaces. Other pathways affected by Scx knockdown included increased oxidative phosphorylation and diseases caused by endoplasmic reticular stress, pointing to a larger role for Scx in maintaining tenocyte homeostasis.

**Conclusions:**

Our study identifies several novel roles for Scx in adult tenocytes, which suggest that Scx facilitates mechanosensing by regulating the expression of several mechanosensitive focal adhesion proteins. Furthermore, we identified a number of other pathways and targets affected by Scx knockdown that have the potential to elucidate the role that tenocytes may play in the development of degenerative tendinopathy.

**Electronic supplementary material:**

The online version of this article (10.1186/s12860-018-0166-z) contains supplementary material, which is available to authorized users.

## Background

Musculoskeletal injuries are common and affect people of all ages, fitness levels, and socioeconomic groups, as well as many animal species, including horses and dogs [1, 2]. Tendon and ligament injuries in particular account for a significant percentage of musculoskeletal injuries each year, with this number expected to rise along with an increasingly sedentary and aging population [1]. Because dense collagenous tissues such as tendon and ligament are slow to heal and the natural healing process often results in the formation of scar tissue, these injuries are particularly problematic [3]. The inability to regain normal tissue structure and mechanical properties often leads to tissue degeneration and chronic reinjury. Despite the frequency at which these injuries occur, and the associated loss of function and productivity they engender, there are few effective treatments available [4]. Tendinopathies are particularly difficult to treat due to their chronic and degenerative nature, which no current treatments are able to adequately resolve. This lack of treatment options is due in part to a lack of understanding of the basic biology of resident tendon cells, called tenocytes. Tenocytes are responsible for the synthesis and maintenance of the normal tendon extracellular matrix architecture in response to physiological load [5]. Decreased production of collagen and upregulation of catabolic enzymes and pro-inflammatory mediators by tenocytes in response to both excessive loading and unloading implicates these cells as a primary driver of degenerative tendinopathy [6, 7]. A better understanding of how tenocytes sense and respond to physical strain could therefore lead to more effective treatments.

Much of the available information regarding tenocyte behavior has been gleaned through investigation of the basic helix-loop-helix transcription factor, scleraxis (Scx). Scx is frequently used as a tendon marker, and is critically involved in both the development of force-transmitting tendons in mice and the tenogenic differentiation of stem cells [8–10]. Mechanical load increases Scx expression [11, 12] and Scx is required for the pro-tenogenic effects of cyclic strain on stem cells [13]. Scx also plays a part in regulating the response to mechanical load in adult mice, with decreased expression following tendon unloading and increased expression in response to physiological load [14, 15]. Taken together, this information demonstrates an important, but not well-characterized, role for Scx in tenocyte mechanotransduction.

To gain better insight into how Scx facilitates tenocyte mechanotransduction, we used small interfering RNA (siRNA) to knock down expression of Scx in adult equine tenocytes and subsequently exposed them to cyclic mechanical load. The resulting transcriptome was sequenced with RNA-sequencing (RNA-seq) technology and compared to that of control tenocytes. We hypothesized that Scx mediates tenocyte mechanotransduction via regulation of a specific subset of previously unidentified, mechanoresponsive genes.

## Methods

### Tendon fibroblast isolation and culture

For the initial transcriptome study, tenocytes were isolated from the superficial digital flexor tendon (SDFT) of a 5-year-old light breed female donated to the Virginia-Maryland College of Veterinary Medicine (VMCVM) for reasons unrelated to this study. All procedures, including tissue harvest, were performed with IACUC approval. Immediately following euthanasia by barbiturate overdose, the tensile region of both SDFT were aseptically excised, stripped of the paratenon, minced into small pieces (2-5 mm), and digested in growth medium (Dulbecco’s modified Eagle’s medium [DMEM; 4.5 g/L glucose], 10% FBS, 2 mM L-glutamine, 50 μg/mL ascorbic acid, 25 mM HEPES, 100 units/mL penicillin, and 100 μg/mL streptomycin) containing 0.075% collagenase type 2 (Worthington Biochemical, Lakewood, NJ) and 0.06 μg/mL α-ketoglutaric acid overnight at 37 °C, 5% CO_2_, and 90% humidity. Cells were strained, pelleted, and plated at 6000/cm^2^ in growth medium. Growth medium was exchanged every 3 days and cells were passaged upon reaching 70% confluence using 0.25% trypsin-EDTA. For cohort validation studies, tenocytes were isolated and passaged in the same manner from the SDFT of 6 additional light breed horses (mean age 5.8 ± 3.3 years; 4 females, 2 castrated males) donated to the VMCVM for reasons unrelated to this study and under IACUC approval. Tenocytes were used at passage 3 for all experiments.

### siRNA and cyclic strain exposure

Tenocytes were transfected (Nucleofector™ system, Lonza, Cologne, Germany) with a siRNA targeting the equine Scx mRNA (Sense: 5’-AGAGAAAGUUGAGCAAGGAtt-3′, Antisense: 5’-UCCUUGCUCAACUUUCUCUgg-3′, GenBank ref. NM_001105150.1; *Silencer*™ Select, Ambion, Life Technologies, Carlsbad, CA) or a non-targeting scramble siRNA control (*Silencer*™ Select Negative Control No. 1, Catalog #4390843, Life Technologies). Transfection efficiency was evaluated using a fluorescein-conjugated scramble siRNA (sc-36,869; Santa Cruz Biotechnology Inc., Dallas, TX) and counting labeled cells by fluorescent microscopy. Cells were resuspended at 1 × 10^6^/100 μL in Nucleofector™ Cell Line Solution V (Lonza) containing 10 nmol siRNA or scramble control, transferred to cuvettes, and nucleofected using the T20 program. Cells were recovered in growth medium for 15 min at 37 °C before plating at 200,000 cells/well on flexible silicone culture plates (UniFlex® Collagen type I coated; Flexcell International, Hillsborough, NC). Cells were allowed to adhere for 24 h before being synchronized in culture medium containing 1% FBS. After 18 h, cells were exposed to cyclic uniaxial strain (1%, 0.5 Hz, 2 h) every 24 h for 3 days. Thirty minutes after completion of the final strain cycle, cells were collected into guanidine isothiocyanate-phenol solution (TRIzol® Reagent, Invitrogen, Carlsbad, CA) for RNA isolation. The experiment was repeated 3 times to generate 6 total samples for sequencing (3 siRNA and 3 scramble controls). Cohort samples for validation by qPCR were generated in the same manner.

### RNA isolation

Total RNA was isolated by column purification according to manufacturer instructions (Direct-zol Microprep, Zymo Research, Irvine, VA) and evaluated both spectrophotometrically for quantity (NanoDrop, Thermo Scientific, Waltham, MA) and by electrophoresis for RNA integrity (Bioanalyzer, Agilent, Santa Clara, CA). RNA from the same samples submitted for RNA-seq and the additional cohort were isolated and converted to cDNA for use in qPCR validation studies (High-Capacity cDNA Reverse Transcription Kit, Applied Biosystems, Foster City, CA).

### Transcriptomic analysis

cDNA library prep and sequencing was performed at the Biocomplexity Institute at Virginia Tech. Total RNA (1 μg per sample) was enriched for polyA RNA (PrepX PolyA mRNA isolation kit, Wafergen, Fremont, CA) and converted to cDNA libraries (PrepX RNA-seq for Illumina Library Kit, Wafergen). Libraries underwent 13 rounds of PCR to generate the final cDNA libraries for sequencing. Individual sample libraries were clustered and sequenced on the Illumina HiSeq 2500 (average of 26.9 million paired end reads for scramble control and 29.3 million for Scx siRNA). Raw sequence data were evaluated for quality (FastQC) [16] and adaptor sequences and low quality reads were removed using Trimmomatic [17] prior to being aligned to the reference genome (EquCab2) using HISAT2 [18] and mapped to known features (Ensembl EquCab2 version 90) using HTSeq [19]. Differential gene expression between scramble control and Scx knockdown samples was determined using DESEQ2 [20]. All fold changes are shown relative to the scramble control and on a log2 scale, unless otherwise stated. Genes with a ± 1.5 log2fold change and an adjusted *p*-value of *p* < 0.05 were used in functional annotation and gene ontology (GO) enrichment analysis of differentially expressed genes using the PANTHER Classification System (version 13.0, http://www.pantherdb.org/) and a false discovery rate of 0.05. KEGG Pathway analysis was performed with the DAVID Bioinformatics Resource (version 6.7, https://david.ncifcrf.gov/) and significance set at *p* < 0.05 as evaluated by modified Fisher Exact test (EASE score). Sequence data generated in this study have been submitted to National Center for Biotechnology Information Gene Expression Omnibus (NCBI GEO, https://www.ncbi.nlm.nih.gov/geo/) under accession number GSE110567. Differential expression analysis and normalized counts for all genes and samples are included as Additional file 1.

### qPCR analysis

Minor groove binding primer-probe sets were purchased (Scx, assay #Ec03818452_s1, Life Technologies) or designed for genes of interest identified by the transcriptomic analysis (Primer Express®, Applied Biosystems; Table 1). All primer-probe sets had an efficiency of > 90% as determined by serial dilution against a known template (TaqMan™ Master Mix, Life Technologies; StepOnePlus™ Real-Time PCR System, Applied Biosystems). Relative gene expression was calculated using the ΔΔCt method and the housekeeping gene glyceraldehyde 3-phosphate dehydrogenase (GAPDH) [21, 22]. Data are shown relative to the average of the scramble control samples for each gene.

**Table 1:** Equine specific primers used for qPCR

Gene	Forward (5′ to 3′)	Reverse (5′ to 3′)	Probe (5′ to 3′)
GAPDH	CAAGTTCCATGGCACAGTCAAG	GGCCTTTCCGTTGATGACAA	CCGAGCACGGGAAG
BCAR1	CCAAGATCTTTGTGGCACACA	CCCGATGAACACCAGCTTGT	CAAATTCGTCATCCTCA
TLN1	GAAGATGAGGCCACCAAAGG	GACCGCCAGTTCCTGACGTA	ACACGGGCCCTGGA
TLN2	CCGTGTCTGACTCCATCAAGAG	TGCCATCAATGGAGTAGTCACACT	TCATCACATCTATCAGAGACAA
FLNB	CCTCGCTGCCACCTGATC	AGCTCCTTTGGTGTCGATGGT	TCCAAGGTGAAGGCC
FLNC	GGGCCAAAGGGCACAGA	ACAGGGTAGTACTCACACTCGAACAC	AGCTGGTGAAGGTGCGA

### Immunofluorescent staining and morphological analysis

Cells were exposed to either the scramble control or Scx-targeting siRNA and plated on collagen type I coated tissue culture polystyrene (TCP) plates in the same manner described above for cells plated on silicone bottom plates. After 3 days in culture, cells were fixed in prewarmed 4% paraformaldehyde in dPBS + 0.3% TritonX-100 for 15 min at room temperature. Differences in cytoskeletal and focal adhesion morphology were investigated using a focal adhesion staining kit following manufacturer’s instructions (FAK100, MilleporeSigma, St. Louis, MO). Five random fields were acquired at 20× magnification per condition for each horse and used for analysis of cell morphological features (CellProfiler version 3.0.0, http://cellprofiler.org/).

### Migration assays

Tenocytes were cultured on silicone bottom plates as described above, or on collagen-coated TCP. Scratches were created using a 200 μL pipette tip 30 min following the end of the final strain cycle. Monolayers were rinsed once with dPBS to remove debris and covered with fresh culture medium containing 1% FBS. Images were taken at 0, 3, 5, 8, and 12 h post scratch formation to monitor cell migration into the scratch area (ImageJ, National Institutes of Health, Bethesda, MD).

### Single cell stiffness measurements by atomic force microscopy

Tenocytes were exposed to Scx siRNA and cultured on collagen-coated TCP as described above. Young’s Modulus (*E*) measurements were obtained using a Veeco BioScope II (Veeco Instruments Inc., Planview NY) equipped with a heated stage and blunted pyramidal silicon nitride cantilever tips (spring constant = 0.06 N m^−^ 1, half open angle = 18°; DNP-10, Bruker Nano Inc., Camarillo, CA). Force-distance curves were captured in contact mode at 1 Hz for a Z-scan distance of 1 μm. To determine Young’s Modulus, raw data were fit to a modified Hertz cone model for up to 10% of the peri-nuclear cell thickness to eliminate any influence from the culture dish using Eqs. (1) and (2)

1 $$ F=k\left(d-{d}_0\right) $$

2 $$ F=\frac{2\tan \alpha }{\pi }\ \left[\frac{E}{1-{v}^2}\right]\ {\delta}^2 $$

where *F* = applied force, *k* = spring constant of the cantilever, *d*_*0*_ = deflection point during cell contact, α = half open angle of the tip, *v* = Poisson’s Ratio (0.5), and δ = indentation [23, 24]. Triplicate force distance curves were collected and averaged for at least 8 cells per condition and repeated for each horse in the cohort (*n* = 7).

### Statistical analysis

All statistical analyses were performed using SAS Studio 3.6 (SAS Institute Inc., Cary, NC). Unpaired Student’s T-Tests were used to assess differences in gene expression, cell stiffness, and morphometric data. Migration assay data were analyzed by mixed model ANOVA with Tukey’s post hoc testing using the PROC GLIMMIX procedure. Model fit was evaluated by examining studentized residual plots. Statistical significance was set at *p* ≤ 0.05. Box and whisker plots represent the median value and 25th and 75th percentiles, with whiskers denoting the minimum and maximum values. All remaining data are shown as mean ± SD.

## Results

### Scx knockdown by siRNA

**Fig. 1:**
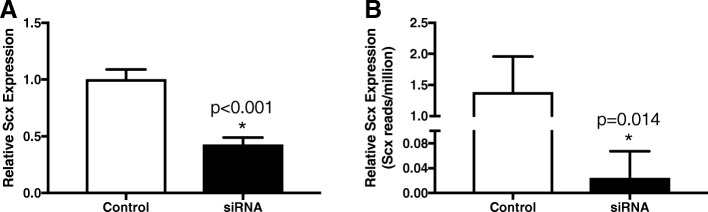
Scleraxis (Scx) transcript knockdown as measured by (**a**) qPCR and (**b**) RNA-seq in sequenced samples. Equine tenocytes exposed to a siRNA targeting Scx for 3 days had decreased expression of Scx mRNA, validating both the effectiveness of the siRNA and the RNA-seq data (*n* = 3)

**Fig. 2:**
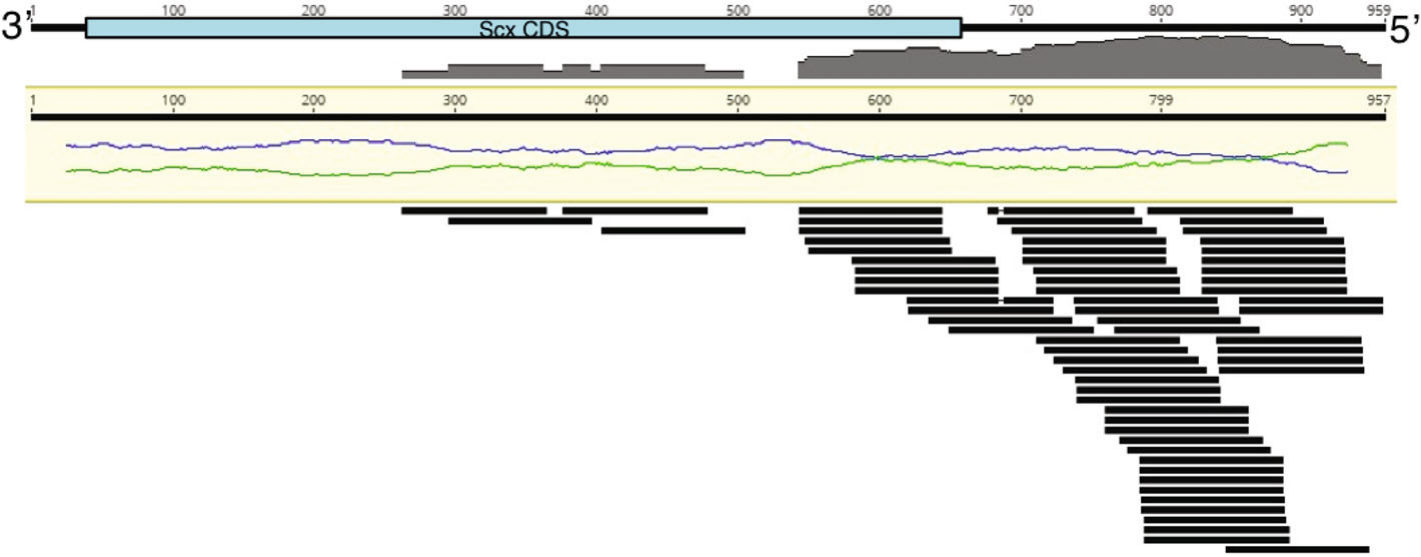
GC bias apparent in reads mapping to scleraxis (Scx) mRNA. Shown are reads from a representative scramble control mapped to the equine Scx mRNA (NM_001105150.1). GC content (blue line) of the reference sequence is shown relative to the AT content (green line). The majority of the reads mapped to the 5’ UTR, which has a GC content of approximately 55%. Few reads mapped to the coding sequence (CDS; ~ 72% GC) and those that did are preferentially located in non-GC biased regions. There were no reads that corresponded to the 3′ end of the Scx CDS, which exhibited the highest GC content (approximately 80%)

Transfection efficiency using the described protocol was > 95% beginning approximately 24 h post-nucleofection and remained > 95% until at least 96 h. Exposure to Scx siRNA using the described protocol resulted in an average knockdown of approximately 57% (*p* < 0.001), as measured by qPCR, in cDNA made from the same samples submitted for RNA-seq (Fig. 1a). As an initial validation of the RNA-seq data, Scx expression was compared between the two methods. The current annotation of the equine genome (EquCab2) does not include Scx as a feature, presumably due to the poor quality of the equine genome upstream of the Scx gene and incomplete information regarding the 3′ end of the Scx coding region. Alignment of RNA-seq reads to the equine Scx mRNA sequence (NM_001105150.1) revealed a substantial GC bias that decreased the total number of mapped reads (Fig. 2). Nevertheless, in samples exposed to Scx siRNA, the number of reads mapping to the Scx mRNA was significantly decreased (*p* = 0.014) compared to the scramble control (Fig. 1b).

### RNA-sequencing and transcriptomic analysis

A total of 11,166 annotated transcripts (out of 26,922) were detected in tenocytes, with 10,231 expressed in both control cells and those exposed to Scx siRNA. An additional 747 genes were expressed in only the control cells and 188 were expressed only in the Scx knockdown cells. The top 25 most highly expressed genes, regardless of Scx knockdown, are shown in Table 2. Vimentin (VIM), a fibroblast marker, was the most highly expressed gene in our dataset. The two major tendon extracellular matrix proteins, collagen types Iα2 (COL1A2) and IIIα1 (COL3A1), were among the most highly expressed genes and were unaffected by Scx knockdown. The small leucine rich proteoglycans decorin (DCN) and lumican (LUM) were both highly expressed and significantly (*p* < 0.001 for both) increased in Scx-depleted tenocytes; however, the fold changes did not meet the inclusion criteria (log2 fold change of >/< 1.5) to be considered for differential expression (LUM = 1.21 log2 fold change, DCN = 0.72 log2 fold change). To further confirm the identity of the cells as tenocytes, data were examined for expression of tendon-enriched genes [25]. Sixty out of 68 of the previously reported tendon-selective genes were present in our dataset (Table 3). The data were also parsed for changes in the expression of other tendon-related genes (Table 4). Expression of collagen types Iα1 (COL1A1) and 5α1 (COL5A1) were decreased, as was expression of the glycoproteins tenascin-C (TNC) and cartilage oligomeric matrix protein (COMP) and the proteoglycan aggrecan (ACAN). Expression of matrix metalloproteinases (MMP) -1 and − 3 was relatively low and unaffected by Scx knockdown. Conversely, MMP-13 expression was increased and MMP-2 expression was decreased in Scx-depleted cells.

**Table 2:** Top 25 most highly expressed genes in equine tendon fibroblasts

Gene ID	ENSEMBL Gene ID	Gene name	Base Mean	Fold Change (log2)	p-adj
VIM	ENSECAG00000004216	vimentin	101,873.95	0.49	0.0846
LUM	ENSECAG00000018248	lumican	93,834.08	1.21	0.0000
EEF1A1	ENSECAG00000020363	eukaryotic translation elongation factor 1 alpha 1	80,865.77	0.35	0.4978
FN1	ENSECAG00000000701	fibronectin 1	76,204.43	− 0.03	0.9131
COL3A1	ENSECAG00000024769	collagen type III alpha 1	71,782.03	−0.28	0.3120
COL1A2	ENSECAG00000024740	collagen type I alpha 2	68,527.63	0.03	0.9444
ACTG1	ENSECAG00000018600	actin gamma 1	67,827.73	0.59	0.1648
DCN	ENSECAG00000020413	decorin	66,957.05	0.72	0.0000
CLU	ENSECAG00000007010	clusterin	60,592.80	0.54	0.3689
CTSK	ENSECAG00000019087	cathepsin K	50,743.84	−0.01	0.9822
PSAP	ENSECAG00000021672	prosaposin	42,671.36	−0.71	0.0123
COL6A3	ENSECAG00000020887	collagen type VI alpha 3 chain	39,357.71	−0.81	0.0000
AHNAK	ENSECAG00000014229	AHNAK nucleoprotein	32,777.16	−1.03	0.0004
TPT1	ENSECAG00000018348	tumor protein, translationally-controlled 1	32,069.02	0.86	0.0000
RPL4	ENSECAG00000023179	ribosomal protein L4	28,412.14	0.55	0.0555
ANXA1	ENSECAG00000015794	annexin A1	28,358.48	1.22	0.0000
HSP90AA1	ENSECAG00000018948	heat shock protein 90 alpha family class A member 1	27,249.39	0.36	0.5410
ASPN	ENSECAG00000007047	asporin	27,102.67	1.44	0.0000
EIF4G2	ENSECAG00000014700	eukaryotic translation initiation factor 4 gamma 2	25,563.63	1.26	0.0000
HSPA8	ENSECAG00000013510	heat shock protein family A (Hsp70) member 8	23,372.98	−0.02	0.9496
HTRA1	ENSECAG00000009990	HtrA serine peptidase 1	23,322.81	−0.18	0.7559
APP	ENSECAG00000021011	amyloid beta precursor protein	21,959.94	−0.08	0.7687
CLTC	ENSECAG00000019077	clathrin heavy chain	21,758.80	0.44	0.1597
CTNNB1	ENSECAG00000006949	catenin beta 1	21,082.51	0.57	0.0512
FAP	ENSECAG00000011790	fibroblast activation protein alpha	20,454.17	1.72	0.0000

**Table 3:** Comparison of genes expressed in current dataset to previously reported tendon-selective genes

Tendon Enriched Gene	Ensembl Gene ID	Mean Counts	Fold Change (log2)	p-adj	Comparison Species
DCN	ENSECAG00000020413	66,957.05	0.72	2.29E-05	Human
ASPN	ENSECAG00000007047	27,102.67	1.44	5.86E-07	Human
THBS1	ENSECAG00000008923	11,775.47	−0.21	5.86E-01	Human
COL12A1	ENSECAG00000025065	10,772.57	−0.34	3.85E-01	Rat
PRRX1	ENSECAG00000008539	3493.07	0.26	2.09E-01	Human
ANKRD12	ENSECAG00000013901	2555.15	0.65	2.34E-01	Human
FBLN1	ENSECAG00000018101	2491.42	0.12	8.31E-01	Human
CCL2	ENSECAG00000023949	2373.29	1.75	1.20E-13	Rat
ATP2B1	ENSECAG00000008450	2263.22	0.57	1.18E-01	Rat
PDE8A	ENSECAG00000007337	2224.57	0.36	2.87E-01	Rat
DTWD1	ENSECAG00000012316	2126.12	1.48	2.68E-07	Rat
BAT2D1 (PRRC2C)	ENSECAG00000016800	1913.66	−0.71	4.85E-03	Human
CREBBP	ENSECAG00000024766	1329.63	−0.75	3.82E-02	Rat
EZR	ENSECAG00000018333	1083.68	−0.78	1.47E-02	Human
IL4RA	ENSECAG00000021525	797.67	−1.28	2.49E-03	Rat
MKX	ENSECAG00000016778	797.26	0.25	5.24E-01	Human
YIPF3	ENSECAG00000016807	739.38	−0.38	5.28E-01	Rat
PSCD3 (CYTH3)	ENSECAG00000025034	615.61	−0.91	9.38E-05	Rat
COMMD7	ENSECAG00000007694	546.66	−0.01	9.86E-01	Rat
LAMA5	ENSECAG00000023274	502.79	−1.81	4.36E-08	Rat
ARSB	ENSECAG00000020847	436.24	−1.37	6.29E-05	Human
SDC1	ENSECAG00000014709	406.86	−0.38	5.54E-01	Rat
FNBP1	ENSECAG00000012905	385.17	−0.50	1.09E-01	Rat
GBA2	ENSECAG00000000580	329.49	−0.76	1.24E-01	Rat
DKK3	ENSECAG00000022804	317.62	−0.50	3.03E-01	Human
RNF41	ENSECAG00000006364	316.63	−0.31	5.79E-01	Rat
LOXL4	ENSECAG00000005573	310.32	−1.18	9.56E-05	Human
MITF	ENSECAG00000005360	282.90	0.19	6.10E-01	Rat
FBXL7	ENSECAG00000005529	269.32	−0.60	3.60E-01	Rat
OAF	ENSECAG00000015986	235.41	−1.17	4.05E-02	Human
IGFBP6	ENSECAG00000019633	235.35	−1.14	3.40E-02	Human
USF1	ENSECAG00000004755	225.09	−1.07	9.76E-04	Rat
NOX4	ENSECAG00000010054	216.23	0.32	4.77E-01	Human
MAB21L1	ENSECAG00000004493	152.16	−0.13	8.19E-01	Rat
CPXM2	ENSECAG00000024631	141.82	−2.11	9.34E-04	Rat
XG	ENSECAG00000000026	126.06	−1.95	7.34E-04	Human
SEMA3B	ENSECAG00000013515	112.02	−0.53	5.97E-01	Rat, Human
EBF1	ENSECAG00000007964	93.12	−0.27	6.99E-01	Rat
WNT5B	ENSECAG00000016516	85.46	−2.02	1.30E-05	Rat
ATF3	ENSECAG00000011486	78.58	0.57	2.26E-01	Rat
GSDMD	ENSECAG00000015005	73.32	−1.81	1.04E-05	Rat
NTRK2	ENSECAG00000011815	50.63	−0.33	6.73E-01	Rat
NOV	ENSECAG00000023039	40.91	−0.24	8.23E-01	Human
AMID (AIFM2)	ENSECAG00000004338	36.15	−1.84	4.66E-04	Rat
C1QTNF2	ENSECAG00000020786	19.91	−0.72	3.43E-01	Human
TNNI3K	ENSECAG00000010595	17.47	0.52	5.59E-01	Human
FGF18	ENSECAG00000019045	17.19	0.17	8.85E-01	Human
IGFBP5	ENSECAG00000013425	16.46	−2.54	8.23E-04	Rat
THBS4	ENSECAG00000019665	14.05	−0.55	5.59E-01	Rat, Human
ELN	ENSECAG00000011106	9.50	−0.74	4.40E-01	Rat
SFRP2	ENSECAG00000017027	8.53	−0.53	6.04E-01	Human
SEPT4	ENSECAG00000020248	5.80	−0.44	6.59E-01	Rat
KERA	ENSECAG00000017668	4.63	0.70	4.66E-01	Human
TRIM29	ENSECAG00000013651	4.12	−0.94	2.95E-01	Human
CCDC3	ENSECAG00000018744	3.46	−0.25	8.05E-01	Human
FKHL18 (FOXS1)	ENSECAG00000001159	2.86	−1.01	2.06E-01	Rat
DPP4	ENSECAG00000017357	2.26	0.36	5.76E-01	Human
MYOC	ENSECAG00000010454	0.16	−0.04	NA	Human
GPR83	ENSECAG00000020552	0.11	−0.04	NA	Human
ANGPTL7	ENSECAG00000010887	0.00	NA	NA	Human
CHODL	ENSECAG00000009963	0.00	NA	NA	Human
CNTN3	ENSECAG00000013575	0.00	NA	NA	Human
ITIH3	ENSECAG00000003355	0.00	NA	NA	Rat
SELE	ENSECAG00000008423	0.00	NA	NA	Rat
SERPINB7	ENSECAG00000024951	0.00	NA	NA	Rat
TNMD	ENSECAG00000018944	0.00	NA	NA	Rat, Human
UTS2R	ENSECAG00000005300	0.00	NA	NA	Rat

**Table 4:** Expression of common tendon-related genes

Gene Name	Ensembl Gene ID	Mean Counts	Fold Change (log2)	p-adj
ACAN	ENSECAG00000007493	304.22	−2.04	2.40E-14
BGN	ENSECAG00000018717	6090.64	−0.38	4.74E-01
COL1A1	ENSECAG00000013693	16,336.22	−1.01	2.78E-04
COL5A1	ENSECAG00000009361	1327.94	−1.52	1.48E-07
COMP	ENSECAG00000000336	694.85	−0.90	2.74E-03
FMOD	ENSECAG00000017864	106.08	−0.66	2.73E-01
MMP1	ENSECAG00000023733	4.30	−0.37	7.17E-01
MMP13	ENSECAG00000005506	4779.30	0.85	2.77E-03
MMP2	ENSECAG00000000953	10,991.67	−0.82	8.55E-03
MMP3	ENSECAG00000000750	72.76	−0.49	5.27E-01
TGFB1	ENSECAG00000011671	318.04	−0.90	1.10E-01
TGFB3	ENSECAG00000015029	2488.60	−0.99	3.81E-04
TNC	ENSECAG00000017433	1383.05	−0.77	1.99E-03

*ACAN* aggrecan, *BGN* biglycan, *COL1A1* collagen type 1α1, *COL5A1* collagen type 5α1, *COMP* cartilage oligomeric matrix protein, *FMOD* fibromodulin, *MMP1* matrix metalloproteinase 1, *MMP13* matrix metalloproteinase 13, *MMP2* matrix metalloproteinase 2, *MMP3* matrix metalloproteinase 3, *TGFB1* transforming growth factor beta 1, *TGFB3* transforming growth factor beta 3, *TNC* tenascin C

Of the 11,166 annotated transcripts detected, 1002 genes met the threshold for differential expression (411 upregulated and 591 downregulated genes in Scx depleted tenocytes compared to controls). GO analysis of differentially expressed genes revealed a number of biological processes affected by Scx knockdown (Table 5). Downregulated genes exhibited significant enrichment in processes involved in cell-matrix adhesion (4.8-fold, *p* = 0.037), transmembrane receptor protein tyrosine kinase signaling pathway (2.8-fold, *p* = 0.046), cell differentiation (2.1-fold, *p* = 0.006), and developmental processes (1.6-fold, *p* = 0.007), among others. Genes upregulated by Scx knockdown showed significant enrichment in processes including oxidative phosphorylation (6.6-fold, *p* = 0.02), mitochondrion organization (5-fold, *p =* 0.009), translation (3.2-fold, *p* = 0.011), and transcription from RNA polymerase II promoter (1.97-fold, *p* = 0.022).

**Table 5:** Gene Ontology (GO) analysis of differentially expressed genes

PANTHER GO-Slim Biological Process		Number of genes	Fold enrichment	FDR
Down-regulated	Cell-matrix adhesion (GO:0007160)	6	4.79	3.7E-02
	Protein folding (GO:0006457)	9	4.00	1.8E-02
	Transmembrane receptor protein tyrosine kinase signaling (GO:0007169)	11	2.77	4.6E-02
	Cytoskeleton organization (GO:0007010)	24	2.33	6.8E-03
	Cell differentiation (GO:0030154)	30	2.17	6.1E-03
	Regulation of phosphate metabolic process (GO:0019220)	27	1.99	2.4E-02
	Organelle organization (GO:0006996)	57	1.86	1.2E-03
	Developmental process (GO:0032502)	64	1.63	6.8E-03
	Cellular component organization (GO:0016043)	77	1.58	5.2E-03
	Phosphate-containing compound metabolic process (GO:0006796)	64	1.53	2.3E-02
	Cellular component organization or biogenesis (GO:0071840)	79	1.51	8.6E-03
	Nitrogen compound metabolic process (GO:0006807)	91	1.39	3.1E-02
	Metabolic process (GO:0008152)	195	1.29	3.2E-03
	Primary metabolic process (GO:0044238)	153	1.26	3.7E-02
Up-regulated	Oxidative phosphorylation (GO:0006119)	5	6.64	2.0E-02
	Mitochondrion organization (GO:0007005)	8	5.00	8.9E-03
	Protein complex biogenesis (GO:0070271)	13	3.37	9.0E-03
	Translation (GO:0006412)	12	3.20	1.1E-02
	Protein complex assembly (GO:0006461)	12	3.12	1.2E-02
	Transcription from RNA polymerase II promoter (GO:0006366)	26	1.97	2.2E-02
	Cellular component biogenesis (GO:0044085)	26	1.93	2.3E-02
	Cellular protein modification process (GO:0006464)	26	1.91	2.4E-02
	Cell cycle (GO:0007049)	23	1.89	4.5E-02
	Biosynthetic process (GO:0009058)	55	1.78	3.5E-03
	Organelle organization (GO:0006996)	35	1.68	3.1E-02
	Protein metabolic process (GO:0019538)	44	1.65	2.2E-02
	Cellular component organization (GO:0016043)	51	1.55	2.5E-02
	Cellular component organization or biogenesis (GO:0071840)	53	1.50	3.3E-02
	Primary metabolic process (GO:0044238)	113	1.37	9.7E-03
	Metabolic process (GO:0008152)	137	1.34	5.7E-03

Differentially expressed genes were overlaid onto KEGG database pathways to further define specific pathways affected by Scx knockdown (Table 6). In agreement with the GO analysis, the extracellular matrix-receptor interaction (3.5-fold, *p =* 0.014) and focal adhesion (2.3-fold, *p* = 0.017) pathways were enriched in genes downregulated by Scx knockdown. Pathways represented by upregulated genes included oxidative phosphorylation (6.4-fold, *p* < 0.001), ribosome (6.4-fold, *p* < 0.001), and several neurodegenerative diseases, including Parkinson’s (6.3-fold, *p* < 0.001), Alzheimer’s (4.4-fold, *p* < 0.001), and Huntington’s disease (3.8-fold, *p* < 0.001).

**Table 6:** Enrichment analysis of KEGG Pathways containing differentially expressed genes

KEGG Pathway		Number of genes	Fold enrichment	*p*-value
Down	ECM-receptor interaction (ecb04512)	7	3.51	1.35E-02
	Neurotrophin signaling pathway (ecb04722)	8	2.54	3.55E-02
	Focal adhesion (ecb04510)	11	2.34	1.74E-02
Up	Oxidative phosphorylation (ecb00190)	14	6.38	1.60E-07
	Parkinson’s disease (ecb05012)	14	6.31	1.80E-07
	Ribosome (ecb03010)	10	6.36	1.95E-05
	Alzheimer’s disease (ecb05010)	13	4.41	2.69E-05
	Huntington’s disease (ecb05016)	12	3.79	2.48E-04
	N-Glycan biosynthesis (ecb00510)	4	4.76	4.93E-02

### Validation of RNA-seq results by qPCR

**Fig. 3:**
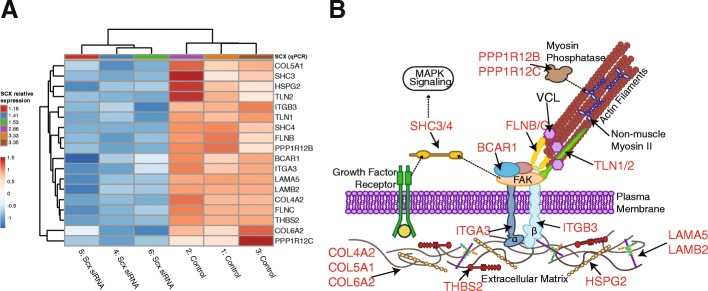
Focal adhesion-related genes downregulated in tenocytes by exposure to scleraxis (Scx) siRNA. Heatmap showing normalized counts of differentially expressed genes related to focal adhesions or extracellular matrix-receptor interactions, clustered by relative Scx expression (as measured by qPCR) and expression pattern (**a**). Location, interactions, and downstream pathways of downregulated genes (**b**). Affected genes are shown in red and unaffected genes in black. Dashed lines indicate interactions between pathway components. COL4A2- collagen type IVα2, COL5A1- collagen type Vα1, COL6A2- collagen type VIα2, THBS4- thrombospondin 4, ITGA3- integrin subunit alpha 3, ITGB3- integrin subunit beta 3, HSPG2- heparin sulfate proteoglycan 2, LAMA5- laminin subunit alpha 5, LAMB2- laminin subunit beta 2, TLN1- talin 1, TLN2- talin 2, FLNB- filamin B, FLNC- filamin C, BCAR1- breast cancer anti-estrogen resistance protein 1, SHC3- SHC adaptor protein 3, SHC4- SHC adaptor protein 4, PPP1R12B- protein phosphatase 1 regulatory subunit 12B, PPP1R12C- protein phosphatase 1 regulatory subunit 12C, VCL- vinculin

To confirm the biological significance of the transcriptomic data, the relationship between Scx knockdown and focal adhesions suggested by the GO and KEGG analyses was further explored. Several differentially expressed, focal adhesion-related genes, were chosen for validation of the sequencing data by qPCR in both the original samples submitted for sequencing and the additional cohort (*n* = 7 horses total). Exposure to Scx siRNA resulted in significantly decreased Scx gene expression compared to controls (Table 7; *p* = 0.036). The remaining genes quantified by qPCR showed comparable downregulation to the RNA-seq results (Table 7), and included several adaptor proteins and those that facilitate force transduction via focal adhesion linkage to the actin cytoskeleton (talin 1 and 2 [TLN1, TLN2], filamin B and B [FLNB, FLNC]; Fig. 3). Other downregulated genes, including breast cancer anti-estrogen resistance protein 1 (BCAR1, also known as p130Cas) and SHC adaptor proteins 3 and 4 (SHC3, SHC4), are key players in the regulation of cell migration by acting as scaffolds for tyrosine kinase-related signaling. Also downregulated in tenocytes exposed to Scx siRNA were several extracellular matrix components (collagen types Vα1, VIα2, and IVα2, laminin subunits α5 and β2, heparan sulfate proteoglycan 2, and thrombospondin 2), integrin subunits α3 and β3, and two regulatory subunits of protein phosphatase 1 (12B and 12C).

**Table 7:** Comparison of gene expression fold changes in RNA-seq data and qPCR results

		Cohort qPCR		
Gene	RNA-seq	Control	Scx siRNA	*p*-value
Scx	–	1.52 ± 1.36	0.41 ± 0.41	0.036
BCAR1	0.35	1.07 ± 0.45	0.71 ± 0.33	0.236
TLN1	0.33	1.07 ± 0.43	0.82 ± 0.45	0.357
TLN2	0.41	1.17 ± 0.71	0.84 ± 0.53	0.105
FLNB	0.39	1.05 ± 0.35	0.80 ± 0.39	0.244
FLNC	0.28	1.07 ± 0.46	0.74 ± 0.37	0.231

*Scx* scleraxis, *BCAR1* Breast cancer anti-estrogen resistance protein 1 (p130CAS), *TLN1* Talin 1, *TLN2* Talin 2, *FLNB* Filamin B, *FLNC* Filamin C

### Effects of Scx knockdown on focal adhesion morphology and cytoskeletal stiffness

**Fig. 4:**
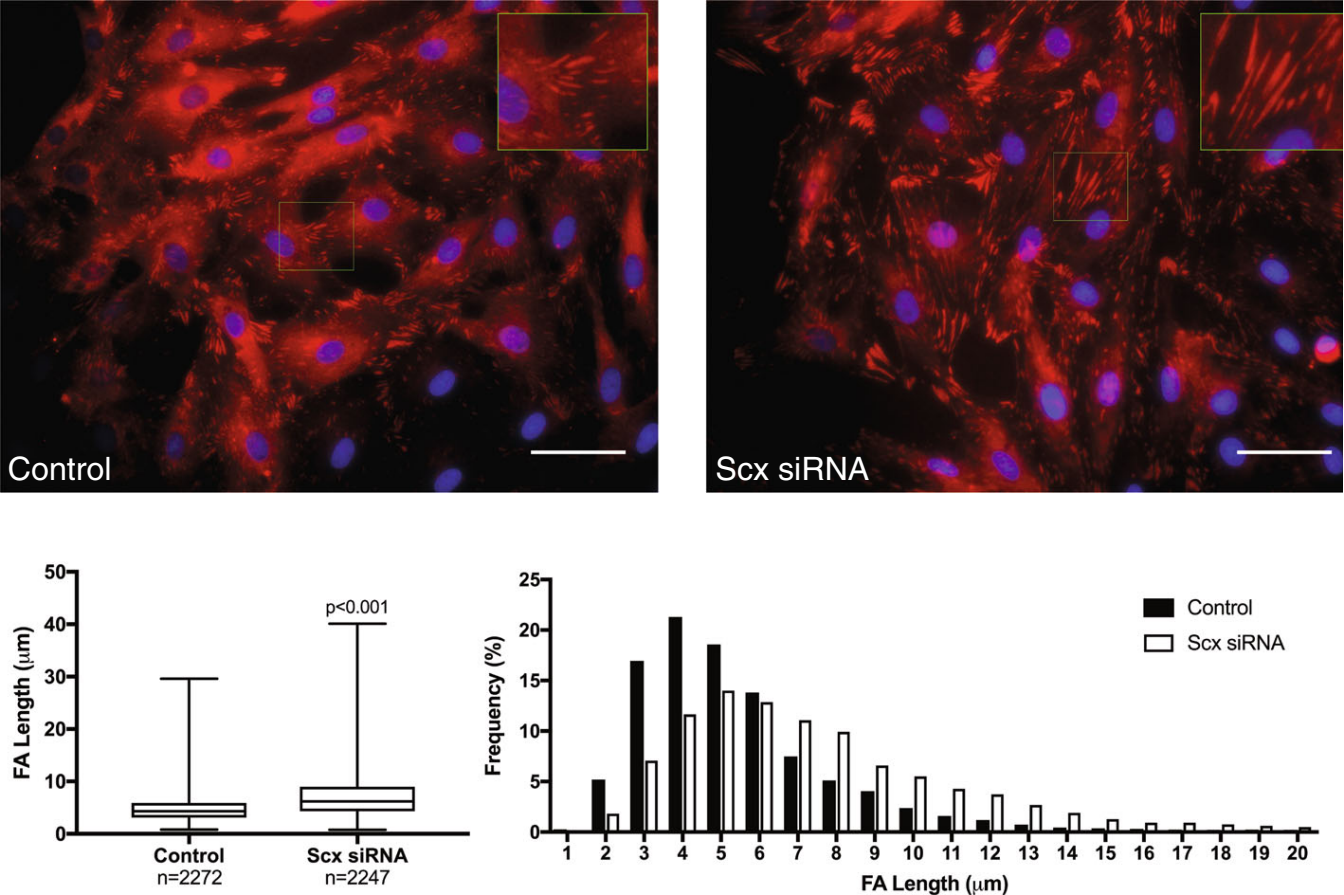
Morphometric analysis of focal adhesions (FA) in tenocytes exposed to Scx siRNA compared to control tenocytes. Representative images of FA staining (top panels; red- vinculin, blue-DAPI. Scale bar = 50 μm). In cells with decreased Scx expression, FA were significantly longer those of control tenocytes (bottom panels)

**Fig. 5:**
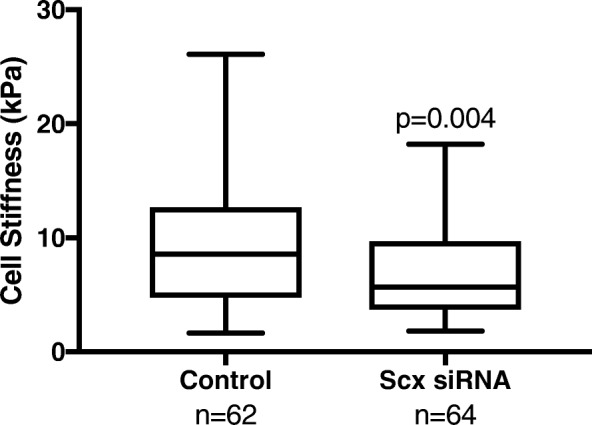
Effect of Scx knockdown on tenocyte stiffness. Young’s modulus for individual cells was determined by atomic force microscopy. Tenocytes exposed to Scx siRNA were significantly softer than control

Changes in cytoskeletal and focal adhesion structure in response to Scx knockdown were examined by immunofluorescent staining for the actin cytoskeleton and vinculin, a protein found in mature focal adhesions that was unaffected by Scx knockdown (Fig. 4). No overt differences were seen in the cytoskeletal organization; however, tenocytes exposed to Scx siRNA had decreased cytosolic staining of vinculin and longer vinculin-containing focal adhesions compared to controls (7.2 ± 4.3 and 4.9 ± 2.6 μm, respectively; *p* < 0.001). In addition, tenocytes exposed to Scx siRNA were approximately 40% softer than control cells (*p* < 0.001), as measured by atomic force microscopy (Fig. 5). Scx knockdown had no significant effect on cell area or nuclear shape (Table 8).

**Table 8:** Cell area and nucleus shape of tenocytes exposed to Scx siRNA and control

	Nuclear Eccentricity	Cell Area (μm^2^)	*n*
Control	0.63 ± 0.12	2183.81 ± 592.02	1623
Scx siRNA	0.63 ± 0.13	2153.26 ± 284.23	1447
*p*-value	0.85	0.06	

### Effect of Scx knockdown on tenocyte migration

**Fig. 6:**
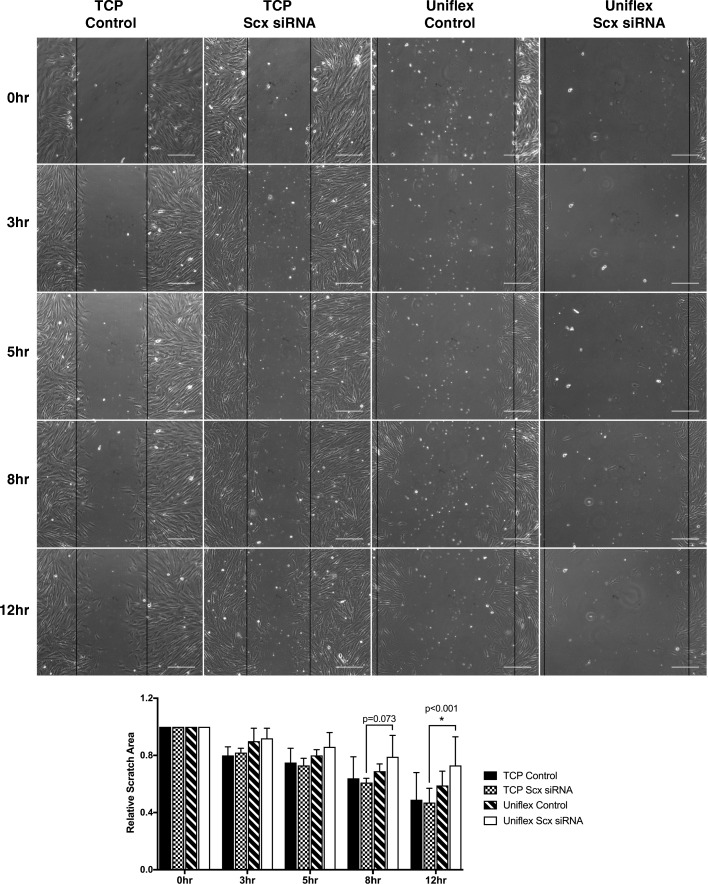
Effects of Scx knockdown on tenocyte migration on substrates of varying stiffness. Tenocytes exposed to Scx siRNA migrated at the same rate as control cells on tissue culture plastic (TCP). In contrast, tenocytes exposed to Scx siRNA migrated slower on silicone substrates (Uniflex plates). Black lines indicate scratch boundaries. Scale bar = 200 μM

There was no difference in cell migration between tenocytes exposed to Scx siRNA and a scramble control siRNA when cultured on collagen-coated TCP (*p* = 0.065; Fig. 6). When migration assays were performed on silicone membranes, however, there was a significant two-way interaction between plate type and Scx knockdown (*p* = 0.025). Though tenocytes tended to migrate slower on the silicone membrane overall, cells exposed to Scx siRNA were significantly slower to migrate on silicone compared to TCP at 12 h post-scratch creation (*p* < 0.001; Fig. 6).

## Discussion

By using a broad, transcriptomic approach followed by biological validation, we identified several novel Scx-mediated processes with important implications in understanding tenocyte behavior. Equine tendon fibroblasts exposed to siRNA targeting Scx were softer, showed an impaired ability to migrate on softer surfaces, and exhibited differences in focal adhesion morphology compared to controls. Together, these findings suggest a potential role and mechanism for Scx in modulating tenocyte mechanotransduction. The results of our study identify interesting new avenues for investigation into tenocyte biology that have the potential to advance our understanding of how physical cues play a role in the development of tendon disorders.

Despite the plethora of information available about the role of Scx in development, few studies have examined the role of Scx in adult tenocytes. A recent in vitro study demonstrated that Scx knockdown in adult equine tenocytes did not affect the expression of common tendon-related genes or the ability to reorganize a 3D collagen matrix [10]. Several groups have reported increased Scx expression in tendons following mechanical load or injury in vivo; however, it appears that the increase in Scx expression is due to proliferation of cells from the tendon periphery and their subsequent migration into the tendon core rather than increased expression by resident tenocytes themselves [15, 26, 27]. In fact, using a murine patellar defect model and Scx-GFP reporter mice, Dyment et al. reported that Scx expression in tenocytes located in the tendon core adjacent to the injured area sharply decreased following injury and remained decreased for at least 7 days before slowly recovering [26]. This suggests that there are at least two Scx-positive cell populations involved in tendon repair and remodeling, though their contributions to overall Scx expression need further clarification. In our study, care was taken to remove the paratenon/epitenon prior to cell isolation in order to examine the effects of Scx knockdown in tenocytes from the tendon parenchyma specifically, though we cannot rule out the presence of endotenon-derived cells.

Scx expression is frequently used as a marker of tenocyte identity, but it is also expressed in other tissues [28, 29]. Tenomodulin (TNMD) and thrombospondin 4 (THBS4) are enriched in tendon compared to other tissues in humans and rats [25]; however, the use of TNMD as a marker of tenocyte identity in horses is less supported, as similar levels of TNMD are found in both tendon and bone [30] and at least one study was unable to detect TNMD expression in normal equine SDFT [31]. In our dataset generated from passaged tenocytes, we observed low levels of THBS4 expression and undetectable levels of TNMD. Despite this, many other tendon selective genes were expressed. Interestingly, a number of these genes, including asporin (ASPN), C-C Motif Chemokine Ligand 2 (CCL2), Laminin Subunit Alpha 5 (LAMA5), and Wnt Family Member 5B (WNT5B), were significantly affected by Scx knockdown. These findings suggest that Scx plays an active role in promoting adult tenocyte identity.

Exposure to Scx siRNA resulted in significantly decreased Scx mRNA as measured by both RNA-seq and qPCR. Scx is not included in the current version (EquCab2) of the equine genome annotation. Therefore, to evaluate Scx expression in our dataset, we mapped sample reads against the Scx mRNA. It is important to note that Scx mRNA is relatively small (957 bp containing two exons), GC-rich (~ 70% overall), and contains regions of stark GC content disparity, ranging from 50 to 80%. As a result, coverage across the Scx mRNA was reduced. GC bias between samples and genes in RNA-seq data are well-documented effects and there are numerous tools to account for these biases in analysis pipelines [32, 33]. Within-transcript bias is also relatively common, but correcting for it is less defined. Use of GC unaware transcript estimation methods can lead to errors in transcript abundance, especially when examining differential isoform expression [34]. In the case of Scx, there are no documented transcript variants; however, care should be taken in evaluating the expression of Scx in transcriptomic studies, as low levels of expression may relate to bias in the sequencing technology and not a biological phenomenon.

Functional annotation and GO analysis showed that Scx knockdown significantly downregulated pathways involved in focal adhesion and extracellular matrix-receptor interactions in our study. Focal adhesions are large protein complexes that form the physical link connecting the cytoskeleton to the extracellular matrix through the binding and activation of transmembrane proteins called integrins [35]. Integrins themselves are direct mechanosensors, and upon activation facilitate the recruitment of many cytosolic proteins to the plasma membrane to form the intracellular portion of the adhesion complex [36]. The protein talin, in particular, anchors the actin cytoskeleton to the focal adhesion by force-dependent interaction with the cytosolic tail of the β3 integrin (ITGB3) [37, 38]. Talin is a critical mechanosensor, and loss of talin impairs cell migration and extracellular substrate sensing [39, 40]. Following talin activation, the cytosolic protein vinculin is recruited to the focal adhesion and interacts with talin to stabilize the complex [41]. Increased presence of vinculin is indicative of decreased focal adhesion turnover and more mature adhesion, which inhibits cell migration [41, 42].

Other proteins recruited in response to integrin binding, including BCAR1 and the SHC adaptor proteins, enable the integration of physical and chemical cues into downstream pathway activation in response to mechanical stimulation. Increased BCAR1 expression correlates with increased invasive potential of cancer cells, and silencing of BCAR1 or SHC3/4 results in decreased migratory ability [43–46]. Expression of both talin isoforms (TLN1 and TLN2), ITGB3, BCAR1, and SHC3/4 were decreased in tenocytes following Scx knockdown in our study. Consistently, tenocytes exposed to Scx siRNA had longer vinculin-containing focal adhesions, indicating decreased focal adhesion turnover. Despite the presence of longer focal adhesions, there was no effect of Scx knockdown on tenocyte migration on TCP. Interestingly, Scx-depleted tenocytes migrated more slowly on softer silicone membranes, whereas control cells were unaffected by the change in substrate stiffness. As many of the genes affected by Scx knockdown function as link proteins at the interface between integrins and the actin cytoskeleton, this substrate stiffness-dependent migration effect could reflect an inability to generate proper cytoskeletal traction.

In further support of a role for Scx in modulating cytoskeletal tension, tenocytes exposed to Scx siRNA exhibited a significant decrease in cytoskeletal stiffness compared to controls. Previous studies have reported that cells alter their cytoskeletal tension in response to environmental changes in order to maintain a predetermined tensional homeostasis [47, 48]. Loss of tensional homeostasis in tenocytes leads to upregulation of MMP-13 and downregulation of COL1A1 [49, 50]. Recovery of cytoskeletal tension in tenocytes occurs through actin-mediated interaction with the local environment [51]. In our study, we observed a similar increase in MMP-13 and decrease in COL1A1 expression, in addition to reduced cytoskeletal stiffness. Inability of the tenocytes to form adequate focal adhesion to actin cytoskeleton connections due to decreased expression of the key adaptor proteins seen in our study (i.e. TLN1/2, FLNB/B, SHC3/4) would impair ability of tenocytes to sense and respond to changes in substrate stiffness and could therefore result in reduced cytoskeletal stiffness.

The relationship between cell stiffness and migratory capacity varies between cell type and disease state. In cancer, decreased cytoskeletal stiffness can be used as an accurate measure of metastatic potential, with cancerous cells being softer than the surrounding healthy cells [52, 53]. In non-cancerous cells, the relationship is less clear. A study by Kole et al. found that in normal 3 T3 fibroblasts, non-migratory cells were significantly softer than migrating cells, indicating that an increase in cytoskeletal stiffness is a prerequisite for directed cell migration [54]. Other studies have shown that disturbed cytoskeletal architecture or connections results in cells with decreased cytoskeletal stiffness and migratory capacity [55, 56]. We observed similar cell behavior in our study, with softer tenocytes migrating more slowly than their stiffer counterparts on softer surfaces. Substrate stiffness, cytoskeletal tension, and migratory capacity are inextricably linked in all cell types. Future studies in tenocytes will help to elucidate the specific relationships and mechanisms involved.

We validated the transcriptomic data by showing that Scx knockdown resulted in slower migrating, softer tenocytes; however, the pathway analysis points to a broader role for Scx in tenocyte homeostasis. Intriguingly, GO term and KEGG pathway analysis showed that a significant number of genes upregulated in response to Scx knockdown are involved in pathways related to neurodegenerative diseases (e.g., Alzheimer’s, Huntington’s, and Parkinson’s). These particular diseases develop, in part, due to dysregulation of the unfolded protein response (UPR), a homeostatic mechanism that has evolved to counter endoplasmic reticular (ER) stress as a result of misfolded proteins [57]. Correspondingly, GO term analysis showed upregulation of many anabolic processes and a concurrent decrease in genes related to protein folding in Scx-depleted cells. ER stress and the UPR are implicated in development of organ fibrosis in heart, lung, and liver disease [58–60]. As the development of fibrotic scar tissue is a major consequence of tendon injury and the main reason for reinjury, this suggested connection between Scx and ER stress in tenocytes warrants further investigation.

## Conclusions

This study is the first to identify specific roles for Scx in adult tenocytes by exposure to siRNA targeting Scx and subsequent RNA-seq interrogation. We confirmed the biological significance of the transcriptomic data by demonstrating that Scx knockdown results in the formation of abnormal focal adhesions, decreased cytoskeletal stiffness, and an impaired ability to migrate on soft substrates. Our data suggest that Scx facilitates tenocyte mechanosensing in part by regulating the expression of several focal adhesion components and genes involved in maintaining cytoskeletal tension. Whether this is the result of direct or indirect gene regulation by Scx remains to be clarified. We also identified other genes and pathways affected by Scx knockdown that point to a larger role for Scx in maintaining adult tenocyte homeostasis. Further exploration of these novel Scx-mediated targets has the potential to advance our understanding of how mechanical strain can lead to tendon injuries.

## Additional file

Additional file 1: Transcriptomic Data. Differential expression analysis and normalized counts for all genes and samples. (XLSX 2345 kb)

## Abbreviations

ACAN: Aggrecan
AMID (AIFM2): Apoptosis Inducing Factor, Mitochondria Associated 2
ANGPTL7: Angiopoietin Like 7
ANKRD12: Ankyrin Repeat Domain 12
ARSB: Arylsultatase B
ASPN: Asporin
ATF3: Activating Transcription Factor 3
ATP2B1: ATPase Plasma Membrane Ca^2+^ Transporting 1
BAT2D1 (PRRC2C): Proline Rich Coiled-Coil 2C
BCAR1: Breast Cancer Anti-Estrogen Resistance Protein 1
C1QTNF2: C1q And TNF Related 2
CCDC3: Coiled-Coil Domain Containing 3
CCL2: C-C Motif Chemokine Ligand 2
CHODL: Chondrolectin
CNTN3: Contactin 3
COL12A1: Collagen Type XII Alpha 1
COL1A1: Collagen Type I Alpha I
COL4A2: Collagen Type IV Alpha 2
COL5A1: Collagen Type V Alpha 1
COL6A2: Collagen Type VI Alpha 2
COMMD7: COMM Domain Containing 7
COMP: Cartilage Oligomeric Matrix Protein
CPXM2: Carboxypeptidase X, M14 Family Member 2
CREBBP: CREB Binding Protein
DCN: Decorin
DKK3: Dickkopf-related Protein 3
DPP4: Dipeptidyl Peptidase 4
DTWD1: DTW Domain Containing 1
EBF1: Early B-cell Factor 1
ELN: Elastin
EZR: Ezrin
FBLN1: Fibulin 1
FBXL7: F-box and Leucine Rich Repeat Protein 7
FGF18: Fibroblast Growth Factor 18
FKHL18 (FOXS1): Forkhead Box S1
FLNB: Filamin B
FLNC: Filamin C
FNBP1: Formin Binding Protein 1
GAPDH: Glyceraldehyde 3-Phosphate Dehydrogenase
GBA2: Flucosylceramidase Beta 2
GO: Gene Ontology
GPR83: G Protein-Coupled Receptor 83
GSDMD: Gasdermin-D
HSPG2: Heparin Sulfate Proteoglycan 2
IGFBP5: Insulin Like Growth Factor Binding Protein 5
IGFBP6: Insulin-like Growth Factor Binding Protein 6
IL4RA: Interleukin-4 Receptor Alpha
ITGA3: Integrin Subunit Alpha 3
ITGB3: Integrin Subunit Beta 3
ITIH3: Inter-Alpha-Trypsin Inhibitor Heavy Chain 3
KERA: Keratocan
LAMA5: Laminin Subunit Alpha 5
LAMB2: Laminin Subunit Beta 2
LOXL4: Lysyl Oxidase Like 4
MAB21L1: Mab-21 Like 1
MITF: Melanogenesis Associated Transcription Factor
MKX: Homeobox Protein Mohawk
MMP: Matrix Metalloproteinase
MYOC: Myocilin
NOV: Nephroblastoma Overexpressed
NOX4: NADPH Oxidase 4
NTRK2: Neurotrophic Receptor Tyrosine Kinase 2
OAF: Out At First Homolog
PDE8A: Phosphodiesterase 8A
PPP1R12B: Protein Phosphatase 1 Regulatory Subunit 12B
PPP1R12C: Protein Phosphatase 1 Regulatory Subunit 12C
PRRX1: Paired Related Homeobox 1
PSCD3 (CYTH3): Cytohesin-3
RNA-seq: RNA-sequencing
RNF41: Ring Finger Protein 41
Scx: Scleraxis
SDC1: Syndecan 1
SDFT: Superficial digital flexor tendon
SELE: Selectin E
SEMA3B: Semaphorin 3B
SEPT4: Septin 4
SERPINB7: Serpin Family B Member 7
SFRP2: Secreted Frizzled Related Protein 2
SHC3: SHC Adaptor Protein 3
SHC4: SHC Adaptor Protein 4
siRNA: small-interfering RNA
TCP: Tissue Culture Polystyrene
THBS1: Thrombospondin 1
THBS2: Thrombospondin 2
THBS4: T; rombospondin 4
TLN1: Talin 1
TLN2: Talin 2
TNC: Tenascin C
TNMD: Tenomodulin
TNNI3K: TNNI3 Interacting Kinase
TRIM29: Tripartite Motif Containing 29
UPR: unfolded protein response
USF1: Upstream Stimulatory Factor 1
UTS2R: Urotensin 2 Receptor
VCL: Vinculin
WNT5B: Wnt Family Member 5B
XG: Xg Blood Group
YIPF3: Yip1 Domain Family Member 3
